# Autophagy pathway upregulation in a human iPSC-derived neuronal model of Cohen syndrome with *VPS13B* missense mutations

**DOI:** 10.1186/s13041-020-00611-7

**Published:** 2020-05-06

**Authors:** You-Kyung Lee, Soo-Kyeong Lee, Suin Choi, Yang Hoon Huh, Ji-Hye Kwak, Yong-Seok Lee, Deok-Jin Jang, Jae-Hyung Lee, Kyungmin Lee, Bong-Kiun Kaang, Chae-Seok Lim, Jin-A Lee

**Affiliations:** 1 Department of Biological Sciences and Biotechnology, Hannam University, 1646 Yuseongdaero, Yuseong-gu, Daejeon, 34054 Korea; 2 Center for Electron Microscopy Research, Korea Basic Science Institute, Daejeon, 34133 Korea; 3 Department of Anatomy, Brain Science & Engineering Institute, Kyungpook National University School of Medicine, Daegu, 41944 Korea; 4 Department of Physiology, Biomedical Sciences, Neuroscience Research Institute, Seoul National University College of Medicine, Seoul, 03080 Korea; 5 Department of Ecological Science, College of Ecology and Environmental Science, Kyungpook National University, Sangju, 37224 Korea; 6 Department of Life and Nanopharmaceutical Sciences, Department of Oral Microbiology, School of Dentistry, Kyung Hee University, Seoul, 02447 Korea; 7 Department of Biological Sciences, College of Natural Sciences, Seoul National University, Seoul, 08826 Korea; 8 Department of Pharmacology, Wonkwang University School of Medicine, 460 Iksan-daero, Iksan, 54538 Korea

**Keywords:** Cohen syndrome, Autophagy, VPS13B, iPSC

## Abstract

Significant clinical symptoms of Cohen syndrome (CS), a rare autosomal recessive disorder, include intellectual disability, facial dysmorphism, postnatal microcephaly, retinal dystrophy, and intermittent neutropenia. CS has been associated with mutations in the *VPS13B* (vacuolar protein sorting 13 homolog B) gene, which regulates vesicle-mediated protein sorting and transport; however, the cellular mechanism underlying CS pathogenesis in patient-derived neurons remains uncertain. This report states that autophagic vacuoles accumulate in CS fibroblasts and the axonal terminals of CS patient-specific induced pluripotent stem cells (CS iPSC)-derived neurons; additionally, autophagic flux was significantly increased in CS-derived neurons compared to control neurons. *VPS13B* knockout HeLa cell lines generated using the CRISPR/Cas9 genome editing system showed significant upregulation of autophagic flux, indicating that *VSP13B* may be associated with autophagy in CS. Transcriptomic analysis focusing on the autophagy pathway revealed that genes associated with autophagosome organization were dysregulated in CS-derived neurons. *ATG4C* is a mammalian ATG4 paralog and a crucial regulatory component of the autophagosome biogenesis/recycling pathway. *ATG4C* was significantly upregulated in CS-derived neurons, indicating that autophagy is upregulated in CS neurons. The autophagy pathway in CS neurons may be associated with the pathophysiology exhibited in the neural network of CS patients.

## Main text

Cohen syndrome (CS) is a rare congenital, neurodevelopmental disorder that manifests clinical features such as obesity, hypotonia, intellectual disabilities, microcephaly, and distinctive facial features [1]. Many people with CS have autosomal recessive mutations in the *VPS13B* gene (also known as *COH1*), which harbors up to 62 exons, and an open reading frame encoding 4022 amino acids and is generally involved in vesicle-mediated trafficking and intracellular protein transport [2]. While *VPS13B* mutant CS mouse models exhibited specific clinical symptoms in CS [3], there is a growing need for pathophysiological studies in human cells derived from CS patient-specific induced pluripotent stem cells (iPSCs). Human skin fibroblasts from CS patients, carrying frameshift or nonsense mutations in *VPS13B*, display a fragmented Golgi complex that leads to disruption of Golgi integrity [4].

**Fig. 1:**
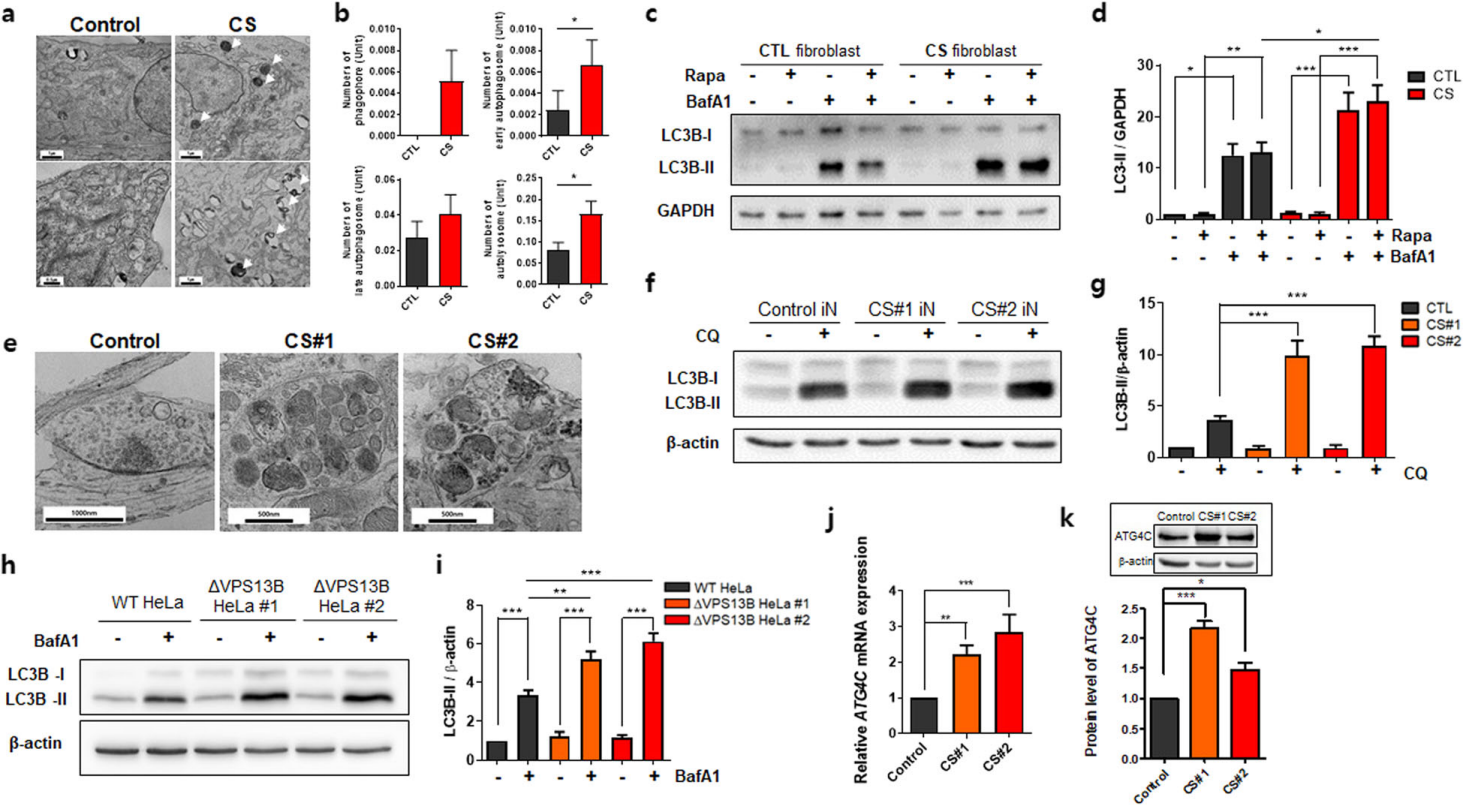
Autophagic flux is upregulated in Cohen syndrome (CS) fibroblasts and CS iPSC-derived neurons. **a** Electron microscopic images of control and CS fibroblasts. **b** Quantitative analysis of EM images. The bar graph indicates the means ± SEM. *, *p* < 0.05 (Student’s t-test). **c** Western blot analysis showing autophagic flux in the presence or absence of a lysosomal inhibitor (bafilomycin A1 [BafA1], 100 nM) with or without rapamycin (100 nM, 4 h) using anti-LC3 or -GAPDH antibodies. **d** Relative expression of LC3-II normalized by GAPDH. The bar graph indicates the means ± SEM from three independent experiments. *, *p* < 0.05; **, *p* < 0.01; ***, *p* < 0.001 (one-way ANOVA followed by Tukey’s Multiple Comparison Test). **e** Electron microscopic images of synaptic regions in control and CS iPSC-derived neurons. **f** Western blot analysis showing autophagic flux in the presence or absence of a lysosomal inhibitor (chloroquine [CQ], 100 nM) using anti-LC3 or -β-actin antibodies. **g** Relative expression of LC3-II normalized by β-actin. The bar graph indicates the means ± SEM from three independent experiments. ***, *p* < 0.001 (one-way ANOVA followed by Tukey’s Multiple Comparison Test). **h** Western blot analysis showing autophagic flux in the presence or absence of a lysosomal inhibitor (BafA1, 100 nM) using anti-LC3 or -β-actin antibodies with WT or *VPS13B* KO HeLa cell (#1, or #2) lysates. **i** Quantification of band intensity of LC3-II or β-actin. The bar graph indicates the means ± SEM from three independent experiments. **, *p* < 0.01; ***, *p* < 0.001 (one-way ANOVA followed by Tukey’s Multiple Comparison Test). **j** The mRNA level of *ATG4C* determined by quantitative RT-PCR. The bar graph indicates the means ± SEM from three independent experiments. **, *p* < 0.01; ***, *p* < 0.001 (one-way ANOVA followed by Tukey’s Multiple Comparison Test). **k** Quantitative analysis of ATG4C expression in CS-derived neurons. (Inset) Western blot images of ATG4C expression in CS-derived neurons. The bar graph indicates the means ± SEM from five independent experiments. *, *p* < 0.05; **, *p* < 0.01 (one-way ANOVA followed by Tukey’s Multiple Comparison Test)

To uncover pathological phenotypes at the ultrastructural level in CS patients with *VPS13B* novel mutations, we performed electron microscopic (EM) analysis on control and CS fibroblasts [5]. There were no gross defects regarding the morphology of the nuclei, ER, or mitochondria (including the Golgi-complex at the ultrastructural level) in CS fibroblasts, however, autophagic vacuoles (AVs), which are a hallmark of autophagy, accumulated tremendously in the cytosol of CS fibroblasts when compared to control fibroblasts (Fig. 1a). The number of early autophagosomes or autolysosomes was significantly increased in CS fibroblasts compared to control fibroblasts (Fig. 1b). To further examine autophagic activity in control and CS fibroblasts, autophagic flux assays were performed in the presence or absence of lysosomal inhibitors (100 nM chloroquine or 100 nM BafA1) for 24 h with or without rapamycin (100 nM). In the presence of lysosomal inhibitors, more accumulation of LC3B-II was found in CS fibroblasts compared to control fibroblasts, indicating that CS fibroblasts exhibit upregulated autophagic flux (Fig. 1c-d).

The autophagy pathway is a conserved cellular degradation and recycling system that relies on lysosomal degradation of superfluous or cytotoxic components and damaged organelles engulfed by autophagic vacuoles in a selective or non-selective manner [6, 7]. Increasing evidence suggests that neuronal presynaptic autophagy in the axon terminal is essential for synaptic maintenance and plasticity [8]. To investigate whether the autophagy pathway is upregulated in CS iPSC-derived neurons, which previously showed synaptic protein (e.g., SV2B) alteration, 4-week old CS neurons were analyzed at the ultrastructural level using EM. Similar to EM results from CS fibroblasts, AVs were accumulated in presynaptic terminals of CS neurons compared to control neurons (Fig. 1e). Autophagic flux was significantly increased in CS neurons compared to control neurons (Fig. 1f-g). These data suggest that autophagy was also upregulated in CS neurons.

Following the previous finding, an examination of how autophagy is upregulated in CS patient-derived fibroblasts and neurons was conducted; previous findings showed that the mRNA level of *VPS13B* was significantly reduced (approximately 50%) in CS fibroblasts with heterogenous missense mutations (c.T1239G [p.Y413X] and c.G10333A [p.V3445M]) compared to control fibroblasts (under revision). Therefore, to clarify the role of *VPS13B* in autophagy, we used the CRISPR/Cas9 genome editing system to disrupt the gene encoding human *VPS13B* in HeLa cells (CRISPR guide RNA sequence [gRNAs] #1: 5′-GGTAATTACCATCAATACTA-3′; gRNAs #2: 5′- AATTGAGGATTCATGTACCA-3′) and validated knockout of *VPS13B* by genomic PCR. Figure 1h-i shows the level of LC3-II in *VPS13B* KO cells was higher in the presence of lysosomal inhibitors than in wild-type HeLa cells, indicating that the loss of *VPS13B* is associated with upregulated autophagic flux, suggesting its potential role in basal autophagy.

To discover autophagy-associated genes responsible for the upregulation of autophagy activity in CS neurons, a transcriptomic analysis for autophagy pathway-related genes in CS neurons and control neurons was implemented. In CS neurons, Gene Ontology analysis showed that two processes (autophagosome organization and macroautophagy) were significant. *MFN2, ATG9B, ATP13A2, KIAA1324, MAP1LC3C,* and *ATG4C* genes were upregulated in CS neurons compared to control neurons. To provide further confirmation, quantitative RT-PCR, and western blot analyses were performed from control and CS neurons and revealed that mRNA and protein levels of *ATG4C* in CS neurons were higher compared to control neurons (Fig. 1j-k). ATG4 is a cysteine protease required for LC3/gamma-aminobutyric acid receptor-associated protein (GABARAP) processing, which allows for the latter to be conjugated to phosphatidylethanolamine on autophagosomal membranes; a key step in autophagosome biogenesis and recycling [9].

Further examination was undertaken to see whether the gene expression of *LC3B* or *GABARAP* was affected in CS neurons. However, there was no significant difference in mRNA expression of *LC3B* or *GABARAP* between control and CS neurons (data not shown). The autophagic flux may be increased, at least partially, by the upregulation of *ATG4C* in CS neurons; however, *VPS13B’s* association with the upregulation of autophagy and CS pathogenesis should be further investigated. The VPS13 family is associated with various human diseases [10]; in particular, a loss of *VPS13A* function disrupts autophagic flux and leads to chorea-acanthocytosis (ChAc), a rare neurodegenerative disease with no known cure. The absence of *VPS13A* or *VPS13B* causes defective or increased autophagic flux; for this reason, autophagy might be a promising therapeutic target for human diseases associated with VPS13 [2, 11].

## Data Availability

All data generated or analyzed during this study are included in this published article.

## Abbreviations

CS: Cohen syndrome
*VPS13B*: vacuolar protein sorting 13 homolog B
ATG: autophagy-related gene
GABARAP: gamma-aminobutyric acid receptor-associated protein
LC3: microtubule-associated proteins 1A/1B light chain 3
CRISPR: clustered regularly interspaced short palindromic repeats
